# A Review of Key Likert Scale Development Advances: 1995–2019

**DOI:** 10.3389/fpsyg.2021.637547

**Published:** 2021-05-04

**Authors:** Andrew T. Jebb, Vincent Ng, Louis Tay

**Affiliations:** ^1^Department of Psychological Sciences, Purdue University, West Lafayette, IN, United States; ^2^Department of Psychology, University of Houston, Houston, TX, United States

**Keywords:** measurement, psychometrics, validation, Likert, reliability, scale development

## Abstract

Developing self-report Likert scales is an essential part of modern psychology. However, it is hard for psychologists to remain apprised of best practices as methodological developments accumulate. To address this, this current paper offers a selective review of advances in Likert scale development that have occurred over the past 25 years. We reviewed six major measurement journals (e.g., *Psychological Methods*, *Educational*, *and Psychological Measurement*) between the years 1995–2019 and identified key advances, ultimately including 40 papers and offering written summaries of each. We supplemented this review with an in-depth discussion of five particular advances: (1) conceptions of construct validity, (2) creating better construct definitions, (3) readability tests for generating items, (4) alternative measures of precision [e.g., coefficient omega and item response theory (IRT) information], and (5) ant colony optimization (ACO) for creating short forms. The Supplementary Material provides further technical details on these advances and offers guidance on software implementation. This paper is intended to be a resource for psychological researchers to be informed about more recent psychometric progress in Likert scale creation.

## Introduction

Psychological data are diverse and range from observations of behavior to face-to-face interviews. However, in modern times, one of the most common measurement methods is the *self-report Likert scale* (Baumeister et al., 2007; Clark and Watson, 2019). Likert scales provide a convenient way to measure unobservable constructs, and published tutorials detailing the process of their development have been highly influential, such as Clark and Watson (1995) and Hinkin (1998) (being cited over 6,500 and 3,000 times, respectively, according to Google scholar).

Notably, however, it has been roughly 25 years since these seminal papers were published, and specific best-practices have changed or evolved since then. Recently, Clark and Watson (2019) gave an update to their 1995 article, integrating some newer topics into a general tutorial of Likert scale creation. However, scale creation—from defining the construct to testing nomological relationships—is such an extensive process that it is challenging for any paper to give full coverage to each of its stages. The authors were quick to note this themselves several times, e.g., “[w]e have space only to raise briefly some key issues” and “unfortunately we do not have the space to do justice to these developments here” (p. 5). Therefore, a contribution to psychology would be a paper that provides a review of advances in Likert scale development since classic tutorials were published. This paper would not be a general tutorial in scale development like Clark and Watson (1995, 2019), Hinkin (1998), or others. Instead, it would focus on more recent advances and serve as a complement to these broader tutorials.

The present paper seeks to serve as such a resource by reviewing developments in Likert scale creation from the past 25 years. However, given that scale development is such an extensive topic, the limitations of this review should be made very explicit. The first limitations are with regard to scope. This is not a review of *psychometrics*, which would be impossibly broad, or advances in self-report *in general*, which would also be unwieldy (e.g., including measurement techniques like implicit measures and forced choice scales). This is a review of the initial development and validation of self-report *Likert scales*. Therefore, we also excluded measurement topics related the *use* self-report scales, like identifying and controlling for response biases.^1^ Although this scope obviously omits many important aspects of measurement, it was necessary to do the review.

Importantly, like Clark and Watson (1995, 2019), Hinkin (1998), this paper was written at the level of the general psychologist, not methodologists, in order to benefit the field of psychology most broadly. This also meant that our scope was to fine articles that were *broad* enough to apply to most cases of Likert scale development. As a result, we omitted articles, for example, that only discussed measuring certain types of constructs [e.g., Haynes and Lench’s (2003) paper on the incremental validation of new clinical measures].

The second major limitation concerns its objectivity. Performing any review of what is “significant” requires, at a point, making subjective judgment calls. The majority of the papers we reviewed were fairly easy to decide on. For example, we included Simms et al. (2019) because they tackled a major Likert scale issue: the ideal number of response options (as well as the comparative performance of visual analog scales). By contrast, we excluded Permut et al. (2019) because their advance was about monitoring the attention of subjects taking surveys online, not about scale development, *per se*. However, other papers were more difficult to decide on. Our method of handling this ambuity is described below, but we do not try claim that subjectivity did not play a part of the review process in some way.

Additionally, (a) we did not survey every single journal where advances may have been published^2^ and (b) articles published after 2019 were not included. Despite all these limitations, this review was still worth performing. Self-report Likert scales are an incredibly dominant source of data in psychology and the social sciences in general. The divide between methodological and substantive literatures—and between methodologists and substantive researchers (Sharpe, 2013)—can increase over time, but they can also be reduced by good communication and dissemination (Sharpe, 2013). The current review is our attempt to bridge, in part, that gap.

**FIGURE 1:**
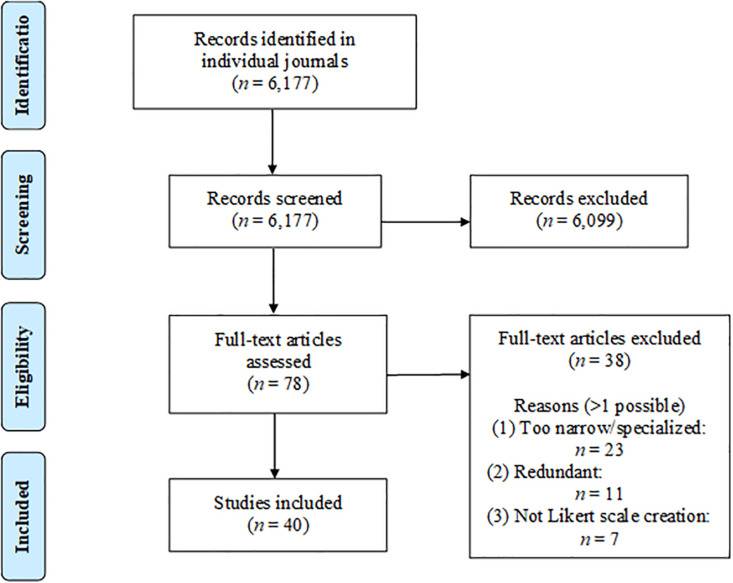
PRISMA flowchart of review process.

To conduct this review, we examined every issue of six major journals related to psychological measurement from January 1995 to December 2019 (inclusive), screening out articles by either title and/or abstract. The full text of any potentially relevant article was reviewed by either the first or second author, and any borderline cases were discussed until a consensus was reached. A PRISMA flowchart of the process is shown in Figure 1. The journals we surveyed were: *Applied Psychological Measurement*, *Psychological Assessment*, *Educational and Psychological Measurement*, *Psychological Methods*, *Advances in Methods and Practices in Psychological Science*, and *Organizational Research Methods*. For inclusion, our criteria were that the advance had to be: (a) related to the creation of self-report Likert scales (seven excluded), (b) broad and significant enough for a general psychological audience (23 excluded), and (c) not superseded or encapsulated by newer developments (11 excluded). The advances we included are shown in Table 1, along with a short descriptive summary of each. Scale developers should not feel compelled to use all of these techniques, just those that contribute to better measurement in their context. More specific contexts (e.g., measuring socially sensitive constructs) can utilize additional resources.

**TABLE 1:** Summary of Likert scale creation developments from 1995–2019.

Aspect of scale development	Summaries of methods
**Conceptions of construct validity**	
Two definitions of validity	See Section 1: “Conceptualizing Construct Validity” Key papers: Borsboom et al. (2004) and Messick (1989)
Validity is “one”	See Section 1: “Conceptualizing Construct Validity” Key paper: Newton and Shaw (2013)
Construct validity since Cronbach and Meehl (1955)	Smith (2005) The author reviews construct validity developments in the previous 50 years since Cronbach and Meehl (1955). The paper begins with developments in philosophy of science and then centers on a five-step model of construct validation, from carefully specifying the target constructs, to revising one’s theory and constructs. Also included is a critical review of several more recent statistical approaches for testing validity (e.g., methods for multitrait/multimethod matrices, generalizability theory).
**Defining constructs**	
Developing clear definitions	See Section 2: “Creating Clearer Construct Definitions” Key paper: Podsakoff et al. (2016)
Specifying the latent continuum	See Section 2: “Creating Clearer Construct Definitions” Key paper: Tay and Jebb (2018)
**Creating scale items**	
Readability tests	See Section 3: “Readability Tests for Items” Key paper: Calderón et al. (2006)
Modern readability measures	Peter et al. (2018) Two newer readability tools can supplement traditional tests for scale items. First, *Coh-Metrix* computes a syntactic simplicity score based on multiple variables (e.g., clauses within sentences, conditionals, negations). Second, the Question Understanding Aid (QUAID) was designed specifically to examine the readability of survey instruments, and can identify potential issues like vague wording, jargon, and working memory overload. Both are freely available at websites listed in the paper.
Respondent comprehension	Hardy and Ford (2014) Good survey data requires that respondents interpret the survey items as the scale developer intended. However, the authors describe how both (a) specific words and (b) the sentences in items can contribute to respondent miscomprehension. The authors provide evidence for this in popular scales and then discuss remedies, such as reducing words and phrases with multiple or vague meanings and collecting qualitative data from respondents about their interpretations of items.
Number of response options and labels	Weng (2004) and Simms et al. (2019) Examining the Big Five Inventory, Simms et al. (2019) found that more Likert response options resulted in higher internal consistency and test-retest reliability (but not convergent validity). These benefits stopped after six response options, and 0–1,000 visual analog scales did not show benefits, either. Including (or removing) a middle point (e.g., “neither agree nor disagree”) did not show any psychometric effects. Weng (2004) also found higher internal consistency and test-retest reliability when all response options had labels compared to when only endpoints of the scale had labels.
Item format	Zhang and Savalei (2016) The authors further research on the expanded scale format as a way to gain the benefit of including reverse worded items (i.e., controlling for acquiescence bias) in a scale without the common downside (i.e., introducing method variance into scores leading to method factor emergence). Each Likert-type item has their response options turned into a set of statements; respondents select one statement from each set.
Item stability	Knowles and Condon (2000) The stability of item properties should not be assumed when it is placed in different testing contexts. There are available methods from classical test theory, factor analysis, and item response theory to examine the stability of items when applied to new conditions or test revisions.
Presentation of items in blocks	Weijters et al. (2014) When putting a survey together, there are many ways to present the scale items. For instance, items from different scales can all be randomized and presented in the same block, or each scale can be given its own block. The authors showed the effects of splitting a unidimensional scale into two blocks with other scales administered in between. Scale items in different blocks had lower intercorrelations, and two factors emerged that corresponded to the two blocks. The authors recommend that assessments of discriminant validity should be mindful of scale presentation and that how scales are presented in surveys should be consistently reported.
**Content validation**	
Guidelines for reporting	Colquitt et al. (2019) Two common methods for content validation are reviewed and compared: Anderson and Gerbing (1991) and Hinkin and Tracey (1999). Both approaches ask subjects to rate how well each proposed item matches the construct definition, as well as the definitions of similar constructs. The authors also offer several new statistics for indexing content validity, provide standards for conducting content validation (e.g., participant instructions, scale anchors), and norms for evaluating these statistics.
Guidelines for assessment	Haynes et al. (1995) Provides an overview of content validation and its issues (e.g., how it can change over time if the construct changes). The authors also provide guidelines for assessing content validity, such as using multiple judges of scales, examining the proportionality of item content in scales, and using subsequent psychometric analyses to indicate the degree of evidence for content coverage.
Consulting focus groups	Vogt et al. (2004) Communicating with the target population is valuable in content validation but is rarely done. One method to do this is to use focus groups, moderator-facilitated discussions that generate qualitative data. This technique can (a) identify the important areas of a construct’s domain, (b) identify appropriate wordings for items, and (c) corroborate or revise conceptualization of the target construct.
Analyzing rating/matching data As item similarity data	Li and Sireci (2013) The authors argue that, compared to traditional content validation ratings/matching data, item similarity ratings are (a) less affected by social desirability and expectancy biases because no content categories are offered and (b) can provide more information about how items group together in multidimensional space. However, having subject matter experts engage in pairwise item similarity comparisons is labor-intensive. The authors offer an innovative method of dummy coding traditional content validation ratings/matching data to essentially derive item similarity data, which is conducive to multidimensional scaling.
**Conducting pilot studies**	
Sample size considerations	Johanson and Brooks (2010) Provides a cost-benefit analysis of increasing sample size relative to decreasing confidence intervals in correlation, proportion, and internal consistency estimates (i.e., coefficient alpha). Found that most reductions in confidence intervals occurred at sample sizes between 24 and 36.
**Measurement precision**	
Limits of reliability coefficients	Cronbach and Shavelson (2004) Although coefficient alpha is the most widely used index of measurement precision, the authors argue that any coefficient is a crude marker that lacks the nuance necessary to support interpretations in current assessment practice. Instead, they detail a reliability analysis approach whereby observed score variance is decomposed into population (or true score), item, and residual variance, the latter two of which comprise error variance. The authors argue that the standard error of measurement should be reported along with all variance components rather than a coefficient. Given that testing applications often use cut scores, the standard error of measurement offers an intuitive understanding to all stakeholders regarding the precision of each score when making decisions based on absolute rather than comparative standing.
Omega/alternatives to alpha	See section 4: “Alternative Estimates of Measurement Precision” Key paper: McNeish (2018)
	Zhang and Yuan (2016) Both coefficient alpha and omega are often estimated using a sample covariance matrix, and traditional estimation methods are likely biased by outliers and missing observations in the data. The authors offer a software package in the R statistical computing language that allows for estimates of both alpha and omega that are robust against outliers and missing data.
Confidence intervals	Kelley and Pornprasertmanit (2016) Because psychologists are interested in the reliability of the population, not just the sample, estimates should be accompanied by confidence intervals. The authors review the many methods for computing these confidence intervals and run simulations comparing their efficacies. Ultimately, they recommend using hierarchical omega as a reliability estimator and bootstrapped confidence intervals, all of which can be computed in R using the ci.reliability() function of the MBESS package (Kelley, 2016).
IRT Information	See section 4: “Alternative Estimates of Measurement Precision” Key paper: Reise et al. (2005)
Controlling for transient error	Green (2003) and Schmidt et al. (2003) Whereas random response error comes from factors that vary moment-to-moment (e.g., variations in attention), transient errors come from factors that differ only across testing occasions (e.g., mood). Because coefficient alpha is computed from a single time point, it cannot correct for transient error and may overestimate reliability. Both articles provide an alternative reliability statistic that controls for transient error, test-retest alpha (Green, 2003), and the coefficient of equivalence and stability (Schmidt et al., 2003).
Test-retest reliability	DeSimone (2015) Test-retest correlations between scale scores are limited for assessing temporal stability. The author introduces several new statistical approaches: (a) computing test-retest correlations among individual scale items, (b) comparing the stability of interitem correlations (*SRMR*_*TC*_) and component loadings (*CL*_*TC*_), and (c) assessing the scale instability that is due to respondents (*D^2^_*pct*_*) rather than scale itself.
	Barchard (2012) Test-retest correlations do not capture absolute agreement between scores and can mislead about consistency. The author discusses several statistics for test-retest reliability based on absolute agreement: the *root mean square difference* [RMSD(A,1)] and *concordance correlation coefficient* [CCC(A,1)]. These measures are used in other scientific fields (e.g., biology, genetics) but not in psychology, and a supplemental Excel sheet for calculation is provided.
Item-level reliability	Zijlmans et al. (2018) Reliability is typically calculated for entire scales but can also be computed for individual items. This can help identify unreliable items for removal. The authors investigate four methods for calculating item-level reliability and find that the correction for attenuation and Molenaar–Sijtsma methods performed best, estimating item reliability with very little bias and a reasonable amount of variability.
**Assessing factor structure**	
Factor analysis practices	Sellbom and Tellegen (2019) The authors provide a timely review of the issues and “pitfalls” in current factor analysis practices in psychology. Guidance is provided for (a) selecting proper indicators (e.g., analyzing item distributions, parceling), (b) estimation (e.g., alternatives to maximum likelihood), and (c) model evaluation and comparison. The authors conclude with a discussion of two alternatives to traditional factor analysis: exploratory structural equation modeling and bifactor modeling.
Exploratory factor analysis	Henson and Roberts (2006) The authors briefly review four main decisions to be made when conducting exploratory factor analysis. Then they offer seven best practice recommendations for reporting how an exploratory factor analysis was conducted after reviewing reporting deficiencies found in four journals.
Exploratory factor analysis for scale revision	Reise et al. (2000) The authors provide guidance on EFA procedures when revising a scale. Specifically, they offer guidance on (a) introducing new items, (b) sample selection, (c) factor extraction, (d) factor rotation, and (e) evaluating the revised scale. However, researchers first need to articulate why the revision is needed and pinpoint where the construct resides in the conceptual hierarchy.
Cluster analysis for dimensionality	Cooksey and Soutar (2006) The authors revive Revelle’s (1978) ICLUST clustering technique as a way to explore the dimensional structure of scale items. The end product is a tree-like graphic that represents the relations among the scale items. The authors claim this method is useful compared to alternatives (e.g., tables of factor loadings).
Unidimensionality	Raykov and Pohl (2013) Some measures may not demonstrate unidimensionality when assessed by fitting a one-factor model to the data due to method or substantive specific factors. This article aims to offer a way to estimate how much of the observed variance in the overall instrument is predominantly explained by a common factor and can thus be treated as essentially homogenous. Mplus and R code are provided to create point and interval estimates for variance explained by both common and specific factors to calculate the difference of these proportions.
	Ferrando and Lorenzo-Seva (2019) Measures are often intended to be unidimensional, but obtained data are found to be better described by multiple correlated factors (or vice versa). Standard goodness of fit assessments (a) are arguably insufficient to adjudicate on which solution is most accurate and (b) only use internal (i.e., item score) information. The authors propose the idea of using external variables (e.g., criteria) to provide evidence for unidimensionality. A procedure to derive (a) primary factor score estimates and then (b) a second-order factor score estimate is described and finally (c) criteria are regressed on them. Lack of differential or incremental prediction of criteria by primary factor score estimates beyond second-order factor score estimates would suggest evidence for unidimensionality.
	Ferrando and Lorenzo-Seva (2018) The authors introduce a program to allow determination of construct replicability, degree of factor indeterminacy and reliability of factor score estimates and explained common variance as an index of unidimensionality. In turn, this has implications for deriving individual scores (i.e., factor score estimates) using exploratory rather than confirmatory factor analysis, the latter of which they argue has the unrealistic assumption of simple structure.
Influence of item wording	McPherson and Mohr (2005) Including both positively- and negatively-worded items in scales is often done but can produce artifactual factors in dimensionality assessments. The authors show that items with more extreme wording (e.g., “I’m always optimistic about the future” vs. “I’m usually optimistic about the future”) can result in greater multidimensionality for the same target construct. The authors recommend that scale developers exercise awareness of these issues and provide recommendations.
**Creating short forms**	
Using IRT information	See section 4: “Alternative Estimates of Measurement Precision” Key paper: Edelen and Reeve (2007)
Ant colony optimization	See section 5: “Maximizing Validity in Short Forms Using Ant Colony Optimization” Key paper: Leite et al. (2008)
**Empirical relations with variables (e.g., nomological network, criterion-related validity)**	
Construct proliferation	Shaffer et al. (2016) Constructs proliferate when discriminant validity is not sufficiently tested. This can happen when (a) important pre-existing constructs are left out of the test or (b) measurement error falsely implies distinct constructs by artificially lowering observed correlations. Remedies for this include (a) making sure all relevant pre-existing constructs have been included, (b) using statistical techniques that account for measurement error (CFA, coefficient of equivalence and stability), and (c) carefully interpreting the results of discriminant validation tests.
	Raykov et al. (2016) The authors challenge the traditional way of assessing construct “congruence” or redundancy by simply fitting a one-factor model to data from measures purportedly measuring two constructs and examining overall fit. Instead, they recommend comparing nested models, where one- and two-factor solutions are fitted and corrected chi-square difference tests are conducted. The authors note that how finding evidence for construct congruence should be interpreted should be left to subject matter experts in that substantive domain.
Incremental validation	Smith et al. (2003) The authors discuss five principles of incremental validation pertinent to scale construction: “(a) careful, precise articulation of each element or facet within the content domain; (b) reliable measurement of each facet through use of multiple, alternate-form items; (c) examination of incremental validity at the facet level rather than the broad construct level; (d) use of items that represent single facets rather than combinations of facets; and (e) empirical examination of whether there is a broad construct or a combination of separate constructs” (p. 467).
	Hunsley and Meyer (2003) The authors review theoretical, design, and statistical issues when conducting incremental validation. Of key importance is the choice of criterion. The criterion should be reliable, and researchers should also be wary of the variety of methodological artifacts that can influence incremental validation results (e.g., criterion contamination, “source overlap”).

**FIGURE 2:**
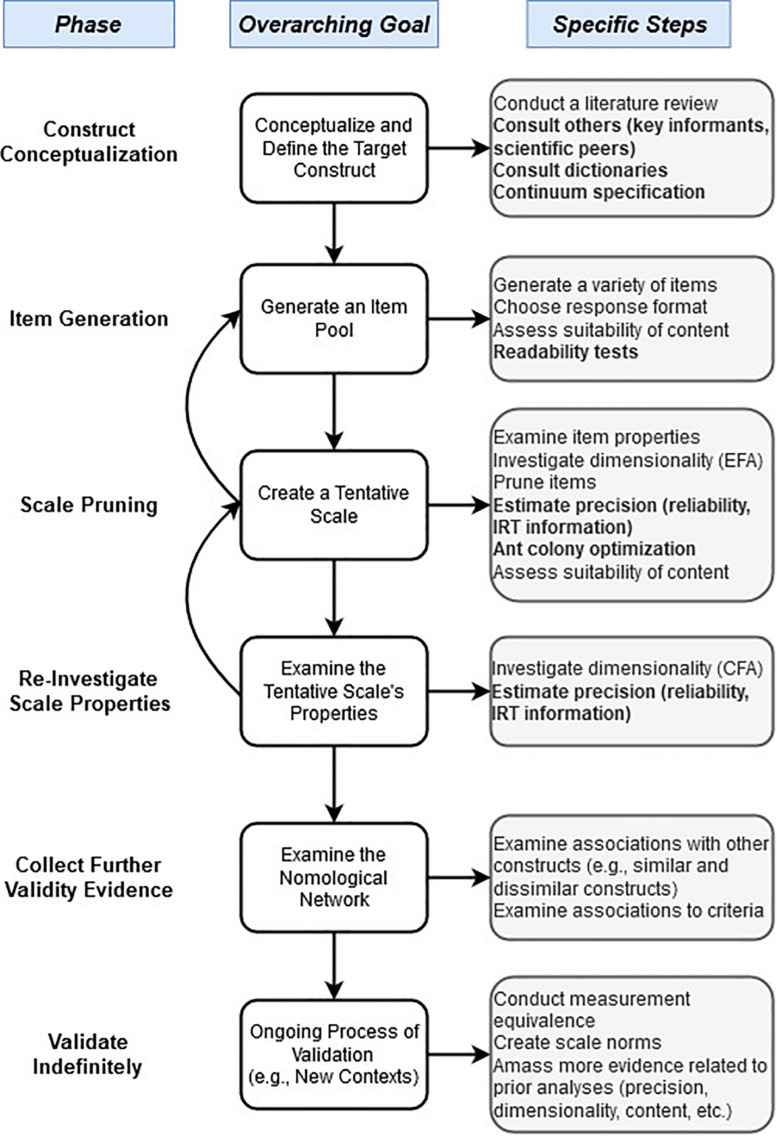
Schematic diagram of Likert scale development (with advances in current paper, bolded).

To supplement this literature review, the remainder of the paper provides a more in-depth discussion of *five* of these advances that span a range of topics. These were chosen due to their importance, uniqueness, or ease-of-use, and lack of general coverage in classic scale creation papers. These are: (1) conceptualizations of construct validity, (2) approaches for creating more precise construct definitions, (3) readability tests for generating items, (4) alternative measures of precision (e.g., coefficient omega), and (5) ant colony optimization (ACO) for creating short forms. These developments are presented in roughly the order of what stage they occur in the process of scale creation, a schematic diagram of which is shown in Figure 2.

## Conceptualizing Construct Validity

### Two Views of Validity

Psychologists recognize validity as the fundamental concept of psychometrics and one of the most critical aspects of psychological science (Hood, 2009; Cizek, 2012). However, what is “validity?” Despite the widespread agreement about its importance, there is disagreement about how validity should be defined (Newton and Shaw, 2013). In particular, there are two divergent perspectives on the definition. The first major perspective defines validity not as a property of tests but as a property of the *interpretations* of test scores (Messick, 1989; Kane, 1992). This view can be therefore called the interpretation camp (Hood, 2009) or validity as *construct validity* (Cronbach and Meehl, 1955), which is the perspective endorsed by Clark and Watson (1995, 2019) and standards set forth by governing agencies for the North American educational and psychological measurement supracommunity (Newton and Shaw, 2013). Construct validity is based on a synthesis and analysis of the evidence that supports a certain *interpretation* of test scores, so validity is a property of *interpretive inferences about test scores* (Messick, 1989, p. 13), especially interpreting score *meaning* (Messick, 1989, p. 17). Because the context of measurement affects test scores (Messick, 1989, pp. 14–15), the results of any validation effort are conditional upon the context in and group characteristics of the sample with which the studies were done, as are claims of validity drawn from these empirical results (Newton, 2012; Newton and Shaw, 2013).

The other major perspective (Borsboom et al., 2004) revivifies one of the oldest and most intuitive definitions of validity: “…whether or not a test measures what it purports to measure” (Kelley, 1927, p. 14). In other words, on this view, validity is a property of *tests* rather than interpretations. Validity is simply whether or not the statement, “test X measures attribute Y,” is true. To be true, it requires (a) that Y exists and (b) that variations in Y *cause* variations in X (Borsboom et al., 2004). This definition can be called the *test validity* view and finds ample precedent in psychometric texts (Hood, 2009). However, Clark and Watson (2019), citing the *Standards for Educational and Psychological Testing* (American Educational Research Association et al., 2014), reject this conception of validity.

Ultimately, this disagreement does not show any signs of resolving, and interested readers can consult papers that have attempted to integrate or adjudicate on the two views (Lissitz and Samuelson, 2007; Hood, 2009; Cizek, 2012).

### There Aren’t “Types” of Validity; Validity Is “One”

Even though there are stark differences between these two definitions of validity, one thing they do agree on is that there are not different “types” of validity (Newton and Shaw, 2013). Language like “content validity” and “criterion-related validity” is misleading because it implies that their typical analytic procedures produce empirical evidence that does not bear on the central inference of interpreting the score’s meaning (i.e., construct validity; Messick, 1989, pp. 13–14, 17, 19–21). Rather, there is only (construct) validity, and different validation procedures and types of evidence all contribute to making inferences about score meaning (Messick, 1980; Binning and Barrett, 1989; Borsboom et al., 2004).

Despite the agreement that validity is a unitary concept, psychologists seem to disagree in practice; as of 2013, there were 122 distinct subtypes of validity (Newton and Shaw, 2013), many of them named after the fourth edition of the *Standards* that stated that validity-type language was inappropriate (American Educational Research Association et al., 1985). A consequence of speaking this way is that it perpetuates the view (a) that there are independent “types” of validity (b) that entail different analytic procedures to (c) produce corresponding types of evidence that (d) themselves correspond to different categories of inference (Messick, 1989). This is why to even speak of content, construct, and criterion-related “analyses” (e.g., Lawshe, 1985; Landy, 1986; Binning and Barrett, 1989) can be problematic, since this misleads researchers into thinking that these produce distinct kinds of empirical evidence that have a direct, one-to-one correspondence to the three broad categories of inferences with which they are typically associated (Messick, 1989).

However, an analytic procedure traditionally associated with a certain “type” of validity can be used to produce empirical evidence for another “type” of validity not typically associated with it. For instance, showing that the focal construct is empirically discriminable from similar constructs would constitute strong evidence for the inference of discriminability (Messick, 1989). However, the researcher could use analyses typically associated with “criterion and incremental validity” (Sechrest, 1963) to investigate discriminability as well (e.g., Credé et al., 2017). Thus, the key takeaway is to think not of “discriminant validity” or distinct “types” of validity, but to use a wide variety of research designs and statistical analyses to potentially provide evidence that may or may not support a given inference under investigation (e.g., discriminability). This demonstrates that thinking about validity “types” can be unnecessarily restrictive because it misleads researchers into thinking about validity as a fragmented concept (Newton and Shaw, 2013), leading to negative downstream consequences in validation practice.

## Creating Clearer Construct Definitions

### Ensuring Concept Clarity

Defining the construct one is interested in measuring is a foundational part of scale development; failing to do so properly undermines every scientific activity that follows (T. L. Thorndike, 1904; Kelley, 1927; Mackenzie, 2003; Podsakoff et al., 2016). However, there are lingering issues with conceptual clarity in the social sciences. Locke (2012) noted that “As someone who has been reviewing journal articles for more than 30 years, I estimate that about 90% of the submissions I get suffer from problems of conceptual clarity” (p. 146), and Podsakoff et al. (2016) stated that, “it is…obvious that the problem of inadequate conceptual definitions remains an issue for scholars in the organizational, behavioral, and social sciences” (p. 160). To support this effort, we surveyed key papers on construct clarity and integrated their recommendations into Table 2, adding our own comments where appropriate. We cluster this advice into three “aspects” of formulating a construct definition, each of which contains several specific strategies.

**TABLE 2:** Integrative summary of advice for defining constructs.

**Aspect: Consider the construct Strategies:** 1. *Think about the essence of the construct.* Clear scientific definitions stem from a clear personal understanding of what the concept is. Social and psychological constructs are notoriously difficult to define (e.g., “justice,” “terrorism,” and “pornography”). Therefore, researchers must think carefully about answering, “What *is* this phenomenon? What is its essence, its inherent nature?” It is these questions that a definition answers. 2. *Bring the construct back to reality.* A useful question for increasing clarity is, “Where does this construct *concretely* manifest?” Psychological constructs are abstract, but they typically manifest in some concrete way. These “concretes” are often (a) behaviors, (b) feelings, or (c) cognitions. Analyzing these concretes sheds light on the essence of the construct. For example, the psychological construct, “spousal support,” is abstract. However, some of its concretes would be listening to one’s partner or taking care of a household errand, unasked. Analyzing these (and others) can shed valuable insight into the construct’s meaning. 3. *Think about what the construct is not*. A definition states what something is, and this can be clarified by better understanding what it is *not*. Psychologists can, therefore, identify opposing constructs to clarify the meaning of the target construct. For example, exploring what a “lack of spousal support” means (e.g., dismissing the feelings of the partner, failing to help in tasks) can accurately reveal the essence of support. 4. *Compare the construct to similar constructs*. To figure out what makes a construct unique, it is also helpful to look at similar constructs. It is easy to state how a construct is different from very different ones (e.g., spousal support from life satisfaction). Doing the same with a similar construct is more difficult but also more fruitful. Identifying this point of difference will illuminate the subtleties specific to the target. For instance, how is spousal support differentiated from support by a friend? Answering this question is important and helps creates theoretical precision in one’s definition.
**Aspect: Create a formal definition Strategies:** 1. *Use simple language.* Published definitions will be aimed at a scientific audience. However, more complexity and jargon are not necessarily better and can actually be counterproductive to communicating the construct. A useful exercise is to try to create a definition that is as simple as linguistically possible. Much about the target construct can be learned by reducing the language to its simplest form. 2. *Define any necessary subconcepts*. Relying on other concepts for one’s definition is often unavoidable. However, it is important to be clear about what the subconcepts mean. For example, a hypothetical definition of, “ambition,” could be, “the proactive drive to enhance the self.” However, what is a “proactive drive?” And what does it mean to “enhance the self?” This definition demonstrates that having subconcepts can lead to a lack of clarity when they are not well-defined. Therefore, any subconcepts in a definition must themselves be well-understood, or else a lack of clarity is perpetuated. 3. *Consider the definition’s genus and differentia*. Definitions have two parts. The first part specifies the concept as a member of a larger class. This is the “genus” and serves to ground the construct in prior knowledge. The second part is called the “differentia” and specifies what about the concept is new and distinguished from other members of its class. For example, “spousal support” could be defined as “the aid and emotional care provided to one’s spouse.” In this case, the genus is “aid and emotional care,” because this is general behavior, and the differentia is “provided to one’s spouse,” which sets it apart from other forms of support (e.g., friend or co-worker support). Identifying the genus and differentia in one’s working construct definition is a useful way to dissect one’s construct definition. 4. *Keep them short.* Preferably, construct definitions should seek to state only its essential nature and be relatively short. Scholars should be mindful of the distinction between (a) its essential nature and (b) its secondary properties. A definition is focused on the former.
**Aspect: Consult alternative opinions on the definition Strategies:** 1. *Consult dictionaries.* Dictionaries provide lay, rather than scientific, definitions. It can be beneficial to consult these because they will use more straightforward language. 2. *Review scientific literatures*. Often, the same (or a similar) construct may be in multiple literatures. For example, the self-esteem construct can be found in education and psychology. These definitions likely overlap. Where they do overlap can indicate what the construct has as an essential component, and where they do not can point to what a particular definition may be missing. 3. *Consult subject-matter experts, key informants, and/or practitioners*. People familiar or well-studied with the construct can provide key insight into its nature and allow refinement to one’s working definition. This insight can be gained by a variety of methods, such as interviews, gathering retrospective case studies, focus groups, and other qualitative methods. Because many psychological constructs are colloquial concepts (e.g., “spousal support,” “ambition,” “justice”), in many cases, the average layperson can be a key informant. However, this may not be true for more specialized constructs (e.g., clinical constructs). 4. *Enlist feedback from academic peers*. Perspectives from colleagues who do not study that construct can be highly useful because they may see alternatives to the standard thinking about the construct.

*The advice in this table was taken from Mackenzie (2003), Locke (2012), and Podsakoff et al. (2016).*

### Specifying the Latent Continuum

In addition to clearly articulating the concept, there are other parts to defining a psychological construct for empirical measurement. Another recent development demonstrates the importance of incorporating the *latent continuum* in measurement (Tay and Jebb, 2018). Briefly, many psychological concepts like emotion and self-esteem are conceived as having degrees of magnitudes (e.g., “low,” “moderate,” and “high”), and these degrees can be represented by a construct continuum. The continuum was originally a primary focus in early psychological measurement, but the advent of the convenient Likert(-type) scaling (Likert, 1932) pushed it into the background.

However, defining the characteristics of this continuum is needed for proper measurement. For instance, what do the poles (i.e., endpoints) of the construct represent? Is the lower pole its *absence*, or is it the *presence* of an opposing construct (i.e., a *unipolar* or *bipolar* continuum)? And, what do the different continuum degrees actually represent? If the construct is a positive emotion, do they represent the *intensity* of experience or the *frequency* of experience? Quite often, scale developers do not define these aspects but leave them implicit. Tay and Jebb (2018) discuss different problems that can arise from this.

In addition to defining the continuum, there is also the practical issue of fully *operationalizing the continuum* (Tay and Jebb, 2018). This involves ensuring that the whole continuum is well-represented when creating items. It also means being mindful when including reverse-worded items in their scales. These items may measure an *opposite construct*, which is desirable if the construct is bipolar (e.g., positive emotions as including happy and sad), but contaminates measurement if the construct is unipolar (e.g., positive emotions as only including feeling happy). Finally, developers should choose a response format that aligns with whether the continuum has been specified as unipolar or bipolar. For example, the numerical rating of 0–4 typically implies a unipolar scale to the respondent, whereas a −3-to-3 response scale implies a bipolar scale. Verbal labels like “Not at all” to “Extremely” imply unipolarity, whereas formats like “Strongly disagree” to “Strongly agree” imply bipolarity. Tay and Jebb (2018) also discuss operationalizing the continuum with regard to two other issues, assessing dimensionality of the scale and assuming the correct response process.

## Readability Tests for Items

The current psychometric practice is to keep item statements short and simple with language that is familiar to the target respondents (Hinkin, 1998). Instructions like these alleviate readability problems because psychologists are usually good at identifying and revising difficult items. However, professional psychologists also have a much higher degree of education compared to the rest of the population. In the United States, less than 2% of adults have doctorates, and a majority do not have a degree past high school (U.S. Census Bureau, 2014). The average United States adult has an estimated 8th-grade reading level, with 20% of adults falling below a 5th-grade level (Doak et al., 1998). Researchers can probably catch and remove scale items that are extremely verbose (e.g., “I am garrulous”), but items that might not be easily understood by target respondents may slip through the item creation process. Social science samples frequently consist of university students (Henrich et al., 2010), but this subpopulation has a higher reading level than the general population (Baer et al., 2006), and issues that would manifest for other respondents might not be evident when using such samples.

In addition to asking respondents directly (see Parrigon et al., 2017 for an example), another tool to assess readability is to use readability *tests*, which have already been used by scale developers in psychology (e.g., Lubin et al., 1990; Ravens-Sieberer et al., 2014). Readability tests are formulas that score the readability of some piece of writing, often as a function of the number of words per sentence and number of syllables per word. These tests only take seconds to implement and can serve as an additional way to check item language beyond the intuitions of scale developers. When these tests are used, scale items should only be analyzed *individually*, as testing the readability of the whole scale together can hide one or more difficult items. If an item receives a low readability score, the developer can revise the item.

There are many different readability tests available, such as the Flesch Reading Ease test, the Flesch-Kincaid Grade Level Studies test, the Gunning fog index, SMOG index, Automated Readability Index, and Coleman-Liau Index. These operate in much the same way, outputting an estimated grade level based on sentence and word length.

We reviewed their formulas and reviews on the topic (e.g., Benjamin, 2012). At the outset, we state that no statistic is univocally superior to all the others. It is possible to implement several tests and compare the results. However, we recommend the Flesch-Kincaid Grade Level Studies test because it (a) is among the most commonly used, (b) is expressed in grade school levels, and (c) is easily implemented in Microsoft Word. The score indicates what United States grade level the readability is suited. Given average reading grade levels in the United States, researchers can aim for a readability score of 8.0 or below for their items. There are several examples of scale developers using this reading test. Lubin et al. (1990) found that 80% of the Depression Adjective Check Lists was at an eighth-grade reading level. Ravens-Sieberer et al. (2014) used the test to check whether a measure of subjective well-being was suitable for children. As our own exercise, we took three recent instances of scale development in the *Journal of Applied Psychology* and ran readability tests on their items. This analysis is presented in the Supplementary Material.

## Alternative Estimates of Measurement Precision

### Alpha and Omega

A major focus of scale development is demonstrating its reliability, defined formally as the proportion of true score variance to total score variance (Lord and Novick, 1968). The most common estimator of reliability in psychology is *coefficient alpha* (Cronbach, 1951). However, alpha is sometimes a less-than-ideal measure because it assumes that all scale items have the same true score variance (Novick and Lewis, 1967; Sijtsma, 2009; Dunn et al., 2014; McNeish, 2018). Put in terms of latent variable modeling, this means that alpha estimates true reliability only if the factor loadings across items are the same (Graham, 2006),^3^ something that is “rare for psychological scales” (Dunn et al., 2014, p. 409). Violating this assumption makes alpha *underestimate* true reliability. Often, this underestimation may be small, but it will increase for scales with fewer items and with greater differences in population factor loadings (Raykov, 1997; Graham, 2006).

A proposed solution to this is to relax this assumption and adopt the less stringent *congeneric model* of measurement. The most prominent estimator in this group is *coefficient omega* (McDonald, 1999),^4^ which uses a factor model to obtain reliability estimates. Importantly, omega performs at least as well as alpha if alpha’s assumptions hold (Zinbarg et al., 2005). However, one caveat is that the estimator requires a good-fitting factor model for estimation. Omega and its confidence interval can be computed with the psych package in R (for unidimensional scales, the “omega.tot” statistic from the function “omega;” Revelle, 2008). McNeish (2018) provides a software tutorial in R and Excel [see also Dunn et al. (2014) and Revelle and Condon (2019)].

### Reliability vs. IRT Information

Alpha, omega, and other reliability estimators stem from the classical test theory paradigm of measurement, where the focus is on the overall reliability of the psychological scale. The other measurement paradigm, item response theory (IRT), focuses on the “reliability” of the scale at a given level of the latent trait or at the level of the item (DeMars, 2010). In IRT, this is operationalized as *information*_*IRT*_ (Mellenbergh, 1996)^5^.

Although they are analogous concepts, information_*IRT*_ and reliability are different.

Whereas traditional reliability is only assessed at the scale-level, information_*IRT*_ can be assessed at three levels: the response category, item, and test. Information_*IRT*_ is a full mathematical function which shows how the precision changes across latent trait levels. These features translate into several advantages for the scale developer.

First, items can be evaluated for how much precision they have. Items that are not informative can be eliminated in favor of items that are (for a tutorial, see Edelen and Reeve, 2007). Second, the test information function shows how precisely the full scale measures each region of the latent trait. If a certain region is deficient, items can be added to better capture that region (or removed, if the region has been measured enough). Finally, suppose the scale developer is only interested in measuring a certain region of the latent trait range, such as middle-performers or high and low performers. In that case, information_*IRT*_ can help them do so. Further details are provided in the Supplementary Material.

## Maximizing Validity in Short Forms Using Ant Colony Optimization

Increasingly, psychologists wish to use short scales in their work (Leite et al., 2008),^6^ as they reduce respondent time, fatigue, and required financial compensation. To date, the most common approaches aim to maintain *reliability* (Leite et al., 2008; Kruyen et al., 2013) and include retaining items with the highest factor loadings and item-total correlations. However, these strategies can incidentally impair measurement (Janssen et al., 2015; Olaru et al., 2015; Schroeders et al., 2016), as items with higher intercorrelations will usually have more similar content, resulting in less scale content (i.e., the *attenuation paradox*; Loevinger, 1954).

A more recent method for constructing short forms is a computational algorithm called ACO (Dorigo, 1992; Dorigo and Stützle, 2004). Instead of just maximizing reliability, this method can incorporate any number of evaluative criteria, such as associations with variables, factor model fit, and others. When reducing a Big 5 personality scale, Olaru et al. (2015) found that, for a mixture of criteria (e.g., CFA fit indices, latent correlations), ACO either equaled or surpassed the alternative methods for creating short forms, such as maximizing factor loadings, minimizing modification indices, a genetic algorithm, and the PURIFY algorithm (see also Schroeders et al., 2016). Since ACO has been introduced to psychology, it has been used in the creation of real psychological scales for proactive personality and supervisor support (Janssen et al., 2015), psychological situational characteristics (Parrigon et al., 2017), and others (Olaru et al., 2015; Olderbak et al., 2015).

The logic of ACO comes from how ants resolve the problem of determining the shortest path to their hive when they find food (Deneubourg et al., 1983). The ants solve it by (a) randomly sampling different paths toward the food and (b) laying down chemical pheromones that attract other ants. The paths that provide quicker solutions acquire pheromones more rapidly, attracting more ants, and thus more pheromone. Ultimately, a positive feedback loop is created until the ants converge on the best path (the solution).

The ACO algorithm works similarly. When creating a short form of *N* items, ACO first randomly samples *N* items from the full scale (the *N* “paths”). Next, the performance of that short form is evaluated by one or more statistical measures, such as the association with another variable, reliability, and/or factor model fit. Based on these measures, if the sampled items performed well, their probability weight is increased (the amount of “pheromone”). Over repeated iterations, the items that led to good performance will become increasingly weighted for selection, creating a positive feedback loop that eventually converges to a final solution. Thus, ACO, like the ants, does not search and test all possible solutions. Instead, it uses some criterion for evaluating the items and then uses this to update the probability of selecting those items.

ACO is an automated procedure, but this does not mean that researchers should accept its results automatically. Foremost, ACO does not guarantee that the shortened scale has satisfactory content (Kruyen et al., 2013). Therefore, the items that comprise the final scale should always be examined to see if their content is sufficient.

We also strongly recommend that authors using ACO be explicit about the specifications of the algorithm. Authors should always report (a) what criteria they are using to evaluate short form performance and (b) how these are mathematically translated into pheromone weights. Authors should also report all the other relevant details of conducting the algorithm (e.g., the software package, the number of total iterations). In the Supplementary Material, we provide further details and a full R software walkthrough. For more information, the reader can consult additional resources (Marcoulides and Drezner, 2003; Leite et al., 2008; Janssen et al., 2015; Olaru et al., 2015; Schroeders et al., 2016).

## Discussion

Measurement in psychology comes in many forms, and for many constructs, one of the best methods is the psychological Likert scale. A recent review suggests that, in the span of just a few years, dozens of scales are added to the psychological science literature (Colquitt et al., 2019). Thus, psychologists must have a clear understanding of the proper theory and procedures for scale creation. This present article aims to increase this clarity by offering a selective review of Likert scale development advances over the past 25 years. Classic papers delineating the process of Likert scale development have proven immensely useful to the field (Clark and Watson, 1995, 2019; Hinkin, 1998), but it is difficult to do justice to this whole topic in a single paper, especially as methodological developments accumulate.

Though this paper reviewed past work, we end with some notes about the future. As methods progress, they become more sophisticated, but sophistication should not be mistaken for accuracy. This applies even to some of the techniques discussed here, such as ACO, which has crucial limitations (e.g., it depends on what predicted external variable is chosen and requires a subjective examination of sufficient content).

Second, we are concerned with the problem of *construct proliferation*, as are other social scientists (e.g., Shaffer et al., 2016; Colquitt et al., 2019). Solutions to this problem include paying close attention to the constructs that have already been established in the literature, as well as engaging in a critical and honest reflection on whether one’s target construct is meaningfully different. In cases of scale development, the developer should provide sufficient arguments for these two criteria: the construct’s (a) importance and (b) distinctiveness. Although scholars are quite adept at theoretically distinguishing a “new” construct from a prior one (Harter and Schmidt, 2008), empirical methods should only be enlisted after this has been established.

Finally, as psychological theory progresses, it tends to become more complex. One issue with this increasing complexity is the danger of creating incoherent constructs. Borsboom (2005, p. 33) provides an example of a scale with three items: (1) “I would like to be a military leader,” (2) “.10 sqrt (0.05+0.05) = …,” and (3) “I am over six feet tall” (p. 33). Although no common construct exists among these items, the scale can certainly be scored and will probably even be reliable, as the random error variance will be low (Borsboom, 2005). Therefore, measures of such incoherent constructs can display good psychometric properties, and psychologists cannot merely rely on empirical evidence for justifying them. Thus, the challenges of scale development of the present and future are equally empirical and theoretical.

## Author Contributions

LT conceived the idea for the manuscript and provided feedback and editing. AJ conducted most of the literature review and wrote much of the manuscript. VN assisted with the literature review and contributed writing. All authors contributed to the article and approved the submitted version.

## Conflict of Interest

The authors declare that the research was conducted in the absence of any commercial or financial relationships that could be construed as a potential conflict of interest.

## Footnotes

1 We also do not include the topic of measurement invariance, as this is typically done to validate a Likert scale with regard to a *new population*.

2 Nor is it true that just because a paper has been published it is a significant advance. A good example is Westen and Rosenthal’s (2003), two coefficients for quantifying construct validity, which were shown to be severely limited by Smith (2005).

3 Alpha also assumes normal and uncorrelated errors.

4 There are several versions of omega, such as hierarchical omega for multidimensional scales. McNeish (2018) provides an exceptional discussion of alternatives to alpha, including software tutorials in R and Excel.

5 There are two uses of the word “information” used in this section: as the formal IRT statistic and the general, everyday sense of the word (“We don’t have enough information.”). For the technical term, we will use information_*IRT*_, and the latter we will leave simply as “information.”

6 One important distinction is between short *scales* and short *forms*. Short forms are a type of short scales, but of course, not all short scales were taken from a larger measure. In this section, we are concerned with the process of developing a short form from an original scale only.
