# Supplementary material for: A Review of Key Likert Scale Development Advances: 1995–2019
Source: Front Psychol. 2021 May 4;12:637547. doi: 10.3389/fpsyg.2021.637547 (PMC8129175; doi:10.3389/fpsyg.2021.637547)
Supplement: Supplementary file 1 [file Data_Sheet_1.docx]

**Supplemental Material for**

**“A Review of Key Likert Scale Development Advances: 1995 – 2019”**

**Readability Tests for Items**

The formula for the Flesch-Kincaid Grade Level Studies test is:

$$FKGL=\left( 0.39\times ASL \right)+\left( 11.8 \times ASW \right)-15.59$$

where ASL is the average sentence length (by number of words), and ASW is the average number of syllables per word. We also analyzed three recent examples of scale development in the *Journal of Applied Psychology*: the Workplace Status Scale (Djurdjevic et al., 2017), the Workplace Gossip scale (Brady, Brown, & Liang, 2017), and the Leader Affect Questionnaire (Martinko et al., 2018). However, before we summarize the results, we want to emphasize that development of clear items is always something that must be *qualitatively* evaluated by the scale developer. Quantitative methods, like readability scores, can be used to aid this process. Readability tests should therefore not be conceived as “hurdles to overcome” but as heuristic checks to flag potentially difficult items. If the readability score indicates that a certain item is very difficult to read, but there is strong rationale for retaining it in the scale, then it probably should be retained. We take time to emphasize these points because sometimes statistics are taken as absolutes, rather than as quantitative summaries with limitations (Gigerenzer, 2004; Orlitzky, 2011). Readability tests are imperfect and have been critiqued as “surface-level indicators as proxies for complex cognitive processes that take place when reading a text” (Benjamin, 2012, p. 63). Using them as heuristic checks avoids these problems.

The results of our analysis are shown in Table S1. Generally, scale items had good readability scores. However, there were several items that were flagged as potentially being difficult. One item in the Workplace Status scale had a reading grade level of 14 (“I occupy a respected position in my organization.”). Despite a high grade level, the item is fairly clear. However, by substituting synonyms, one can preserve the meaning of the item and bring the item to a 5^th^ grade level (“I hold a respected position where I work”). An item in the Positive Leader Affect Questionnaire was over the 12^th^ grade level (“I feel positively about my supervisor”). However, this item is fairly simple because its high-syllable words (“positively” and “supervisor”) are familiar to respondents. Several more flagged items and possible alternative wordings are provided in Table S1.

Table S1.

Three Psychological Scales and their Reading Difficult Levels

| Djurdjevic et al. (2017) Workplace Status Scale |
| --- |
| 1. I have a great deal of prestige in my organization. 4.8 2. I possess high status in my organization. 9.1 3. I occupy a respected position in my organization. 14.1   **Alternative: I hold a respected position where I work. 5.2**   1. I have a position of prestige in my organization. 7.6 2. I possess a high level of prominence in my organization. 9.6 |
| Brady et al. (2017) Negative Workplace Gossip about Coworkers Scale |
| (Stem) In the last month, how often have you…   1. asked a work colleague if they have a negative impression of something that another co-worker has done 11.8   **Alternative: asked a work colleague if they disliked something that a co-worker did 8.3**   1. questioned a co-worker’s abilities while talking to another work colleague 10.5 2. criticized a co-worker while talking to another work colleague 9.1 3. vented to a work colleague about something that another co-worker has done 10 4. told an unflattering story about a co-worker while talking to another work colleague 11.2   **Alternative: told an unflattering story to a co-worker about another co-worker 9.9** |
| Martinko et al. (2018) Positive Leader Affect Questionnaire |
| 1. I feel positively about my supervisor. 12.3   **Alternative: I feel good about my supervisor. 6.4**   1. I like my supervisor. 6.6 2. I like to work with my supervisor. 4.0 3. I value the relationship I have with my supervisor. 8.9 4. I have been happy with my supervisor. 5.7 |

*Note*: Numbers at the end of each item are its score on the Flesch-Kincaid Grade Level Studies test. Possible revisions to items are bolded.

**Alternative Estimates of Measurement Precision: IRT Information**

The theoretical basis of information_IRT_ is found in latent trait theory. We provide only a very brief introduction on IRT here, and more complete introductions can be found in the articles by Reise, Ainsworth, and Haviland (2005) and Edwards (2009), as well as the full texts by Baker (2001) or DeMars (2010). Broadly speaking, items are included in scales because they contribute information on the target attribute (the “latent trait”). For items with Likert scales, each response category is intended to capture a different level of the latent trait. Individuals with low values will find that response categories above the neutral point do not fit their experiences, and the probability of selecting these will be low (approaching zero for the highest response option). By contrast, response categories below the neutral point will be seen as better matches, and the probability of selecting these will systematically rise. For each response category, the probability of being selected can be depicted as a mathematical function, with the latent trait value on the x-axis, and the probability on the y-axis. These are called “item response functions.” When the function is flat, all respondents, regardless of their level of the trait, have the same probability of selecting that response option. Thus, no information is provided on that trait. When the function becomes steeper, individuals with different trait levels begin to have different probabilities of choosing that response category, and more information is contributed. Thus, the *slope* of the item response function indicates how much information is provided because it indicates how strongly individuals are *discriminated* at each point. Information_IRT_ is a function that can be calculated from this slope. In addition to plotting the item response functions, we can also plot how the slope changes, called *information functions*. The information function of three response categories are presented together in Figure S1A. Note that information_IRT_ is on the y-axis instead of the raw probability.


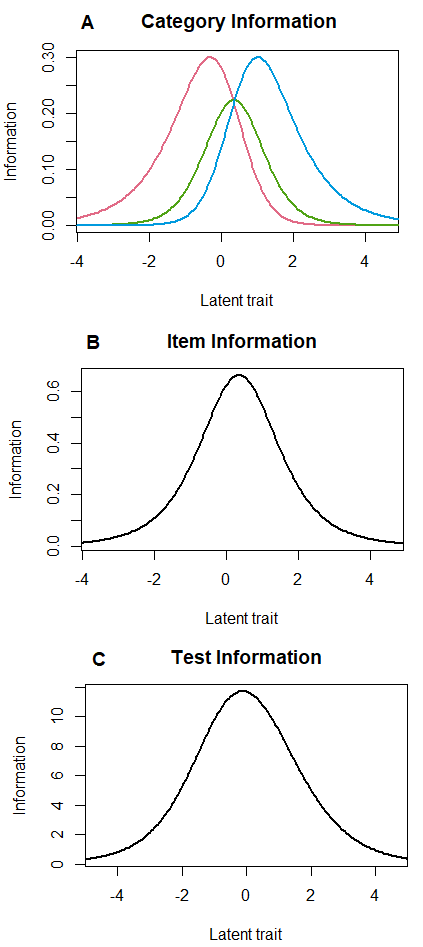


Figure S1. Information functions for category, item, and test.

As can be seen, each category provides information for different levels of the target attribute. These are the *category information functions*. Depending on how many response options there are, these can be summed to yield the *item information function* (shown in Figure S1B). The item information function shows how much information_IRT_ the whole item provides. Furthermore, the information functions for all scale items can be summed to yield the *test information function*, which depicts how much information_IRT_ the whole scale provides. An example test information function is shown in Figure S1C.

For application purposes, we explore one important question: How much information_IRT_ must a scale have to measure the trait adequately? In theory, the more information, the better. However, in practice, the number of items is limited. To answer this question, it is useful to transform information_IRT_ into more intuitive metrics, like the standard error of measurement (O’Connor, 2018). Using the standard error of measurement, one can then determine how much error one is willing to tolerate across the latent continuum. For example, a standard error of 0.20 at a particular trait level means that this is the standard deviation of scores for individuals at that trait level. For interpretation, O’Connor (2018) recommended translating information_IRT_ into traditional reliability values, but values that are “conditional” on levels of the trait (i.e., “conditional reliability values”). Minimum cutoffs for reliability have been recommended at .70 (Hinkin, 1998), which would be an information_IRT_ value of 3.33 at a particular trait level. Nunnally's (1978) recommendation of .80 translates into an information_IRT_ value of 5, and a reliability of .90 means an information_IRT_ value of 10. O’Connor (2018) stated that an information_IRT_ value of 4 could be adequate for research purposes because “attenuation in effect sizes in these cases will be minimal” (p. 1002), but for diagnostic (high-stakes) purposes, it should be much higher, around 10 or above. On pp. 992-993, O’Connor (2018) provides simple formulas for translating information_IRT_ to and from reliability and the standard error of measurement.

**Maximizing Validity in Short Forms Using Ant Colony Optimization**

Although there are different ways to implement ACO (e.g., Janssen et al. 2015), we focus on the approach taken by Leite et al. (2008) because it has proven to be effective, was the first application of ACO to short forms, and is the only software implementation to date that is readily available (through the R “ShortForm” package; Raborn & Leite, 2018).

**Practically, How ACO Works**

To implement ACO in practice, there are two key decisions that must be made. The first is to decide how to define the “performance” of a short form. This is not a simple task because many metrics can be used, such as measures of reliability, CFA model fit indices, or relationships to external variables. In their study, Leite et al. (2008) used two: CFA fit indices (CFI, TLI, and RMSEA) and the value of regression coefficients that predicted a relevant criterion (2-hr postprandial blood sugar) from the five latent factors of the scale they were shortening (a scale for the quality of life scale for diabetes patients). Other studies in psychology have used these metrics and incorporated others, such as reliability, latent correlations between factors, and item difficulties (Janssen et al., 2015; Schroeders et al., 2016).

Once a performance metric has been chosen, the second decision is how to map the values of this metric into how the probability weights will be updated (this is sometimes called the “pheromone function”). For Leite et al. (2008), the probability weight was updated by simply adding the sum of the absolute value of the standardized regression coefficients. In the corresponding ShortForm package, the average of these coefficients is added to the weight. The CFA fit indices influenced the probability weights differently; they acted as a *hurdle* for the short form, such that if the model fit of the original factor structure did not surpass conventional criteria (i.e., CFI < 0.95, TLI < 0.95, and RMSEA > 0.06; Hu & Bentler, 1999), then no probability weight was added to the items. This strategy prevents reinforcing items that lead to a poor model fit and is important for psychological researchers who want to replicate the original factor structure of the parent scale (Smith, Mccarthy, & Anderson, 2000). If all three CFA fit criteria were met, then the weights were updated as described above. It is important to note that other authors have used different approaches for mapping short form performance to the probability weights (e.g., Janssen et al., in press and Schroeders, et al. 2016). For a software tutorial of ACO, see Appendix B.

**R Tutorial for Ant Colony Optimization**

As of this writing, the only readily-available way to conduct ant colony optimization (ACO) for scale reduction is using the ShortForm package in R (Raborn & Leite, 2018). To run, the algorithm requires latent variable modeling. This means one must specify a latent variable model in either the lavaan package in R or MPlus. There are other details of the implementation the reader should know:

- As explained above, given sufficient CFA model fit, ShortForm’s implementation of ACO maximizes the relationship with an external variable. Careful selection of the covariate, then, is essential for ensuring the process works. It also should be said that this is just one kind of data-driven approach, and maximizing this coefficient is just one tool for scale shortening.
- This implementation works with scales with either one or more factors. In the case of a single-factor, the probability weight that is added is just the standardized regression coefficient (and for >1 factors, it is their mean).
- A “best-so-far” strategy is used. Probability weights are not added to the items after each short form is evaluated. Rather, *n* short forms are evaluated, and the best short form from the group is selected. This strategy slightly increases the time to convergence but is worth it because it results in more exploration of the model space.
- After each step, all probability weights of the items are reduced by a certain percentage specified by the user, referred to as “pheromone evaporation” (defaulted to 10%). Pheromone evaporation reduces the inﬂuence of the solutions obtained at earlier stages of the search, when poor-quality solutions are more likely to be selected (Dorigo & Stützle, 2004) and also increases convergence times (With the data that comes with the package, we had troubles with convergence when it was set at 0%).
- A brief reiteration of ACO terminology: probability weights of the items are called “pheromone” levels, and one randomly-selected short form is referred to as an “ant.”

ACO is implemented in the ShortForm R package using one of two functions, antcolony.lavaan or antcolony.mplus. Both of these functions implement ACO in the same way but with different software packages fitting the latent variable model (namely, the lavaan package in R or Mplus). The arguments of these functions either specify (a) the ACO algorithm or (b) how the latent variable model is to be fitted. The two functions have largely the same exact arguments for specifying the ACO algorithm. We only focus only on these arguments in this tutorial. The arguments for the latent variable modeling differ across the two functions because they are specific to either lavaan and MPlus. Readers who want to know more about fitting the latent variable models can see the antcolony.lavaan and antcolony.mplus help pages for further details. The other arguments specifying the ACO algorithm can be grouped into a few categories for simplicity.

***Arguments for Specifying the Short-Form Characteristics***

These arguments depend on the structure of the scale one wants to reduce. One must know (a) the number of original items, (b) the number of factors, and (c) which items load onto which factors.

full = the number of items in the full original scale

factors = the names of the factors, e.g., c(‘visual’, ‘textual’, ‘speed’). The order of the factors

must be preserved in the list.items and i.per.f arguments below

i.per.f = an abbreviation for “items per factor” that states how many items are in the target

short form. This is coded as a vector where each number represents the number of items for the factors listed respectively in the factors argument. To use ACO to find a short form with different numbers of items, the algorithm must be run again separately.

list.items = names the items that should load onto each factor. The argument is a list, where

each vector gives the names of the indicators to each factor found in the factors argument

Example: Suppose that someone has a scale with two factors and 30 items whose variable names are “x1: x30.” Items 1-15 are indicators of the first factor, and items 16-30 are indicators for the second. They would like to reduce the original scale to a 10-item short form with 5 items per subscale. They would specify the following arguments in either the antcolony.lavaan or antcolony.mplus functions:

(…full = 30, factors = c(‘Factor1’, ‘Factor2’), i.per.f = c(5,5), list.items = list(c(‘x1’, ‘x2’, ‘x3’, ‘x4’, ‘x5’, ‘x6’, ‘x7’, ‘x8’, ‘x9’, ‘x10’, ‘x11’, ‘x12’, ‘x13’, ‘x14’, ‘x15’), c(‘x16’, ‘x17’, ‘x18’, ‘x19’, ‘x20’, ‘x21’, ‘x22’, ‘x23’, ‘x24’, ‘x25’, ‘x26’, ‘x27’, ‘x28’, ‘x29’, ‘x30’))…)

***Arguments for Specifying the ACO Algorithm***

ants = the number of short forms that are sampled and evaluated for every iteration of the

algorithm. At each step of the algorithm, the best short form from these is selected and given pheromones (probability weights). A new iteration then starts. The default value is to evaluate 20 short forms per step of the algorithm, and Leite et al. (2008) used 10.

evaporation = a 0-1 value that gives the percentage of the pheromone value that is retained

for each step. It is more useful to think about this argument as specifying how much

pheromone is *lost* between steps of the algorithm (rather than how much is retained). Leite et al. (2008) note that slightly reducing the weight by a small percentage, such as 5%, reduces the influence of earlier, usually poorer, solutions (Dorigo & Stützle, 2004). The default value for the evaporation is 0.9 (for 90%), and Leite et al. (2008) used 95%.

In our experience, specifying the algorithm without evaporation (a value of 1.0) leads to convergence problems.

steps = a number that tells the algorithm when to stop when no better solutions are found. For

X steps, the algorithm stops after there are X iterations with no improvement. The default is 50, and Leite et al. (2008) used 100.

max.run = the maximum total number of ants (short forms) that can be run until the algorithm

stops. The default is 1000, the same used by Leite et al. (2008).

Example: Adding these arguments to the earlier ones:

(…full = 30, factors = c(‘Factor1’, ‘Factor2’), i.per.f = c(5,5), list.items = list(c(‘x1’, ‘x2’, ‘x3’, ‘x4’, ‘x5’, ‘x6’, ‘x7’, ‘x8’, ‘x9’, ‘x10’, ‘x11’, ‘x12’, ‘x13’, ‘x14’, ‘x15’), c(‘x16’, ‘x17’, ‘x18’, ‘x19’, ‘x20’, ‘x21’, ‘x22’, ‘x23’, ‘x24’, ‘x25’, ‘x26’, ‘x27’, ‘x28’, ‘x29’, ‘x30’)), ants = 20, evaporation = 0.9, steps = 100, max.run = 1000)

***Arguments for Defining Acceptable Short Forms***

In order to prevent pheromone from being added to items of bad models, these arguments define a minimum acceptable fit of the latent variable model. Items failing these criteria are ignored. The following arguments specify what these fit criteria. Unlike the prior arguments, these differ between the antcolony.lavaan and antcolony.mplus functions.

For the antcolony.lavaan function:

fit.indices = states, in lavaan format, what fit indices will be used to judge the short forms

fit.statistics.test = the minimum acceptable values of fit for the indices named by the

fit.indices argument. The format must be like how it is shown in the code below.

Example: Adding these arguments to the earlier ones:

(…full = 30, factors = c(‘Factor1’, ‘Factor2’), i.per.f = c(5,5), list.items = list(c(‘x1’, ‘x2’, ‘x3’, ‘x4’, ‘x5’, ‘x6’, ‘x7’, ‘x8’, ‘x9’, ‘x10’, ‘x11’, ‘x12’, ‘x13’, ‘x14’, ‘x15’), c(‘x16’, ‘x17’, ‘x18’, ‘x19’, ‘x20’, ‘x21’, ‘x22’, ‘x23’, ‘x24’, ‘x25’, ‘x26’, ‘x27’, ‘x28’, ‘x29’, ‘x30’)), ants = 20, evaporation = 90, steps = 100, max.run = 1000, fit.indices = c(‘cfi’,’tli’,’rmsea’), (cfi > 0.95)&(tli > 0.95)&(rmsea < 0.06))

For the antcolony.mplus function:

min.CFI = the minimum acceptable CFI value, defaulted to 0.95. Short forms with a CFI less

than this value will receive no pheromone.

min.TLI = the minimum acceptable TLI value, defaulted to 0.95. Short forms with a TLI less

than this value will receive no pheromone.

max.RMSEA = the maximum acceptable RMSEA value, defaulted to 0.06. Short forms with an

RMSEA greater than this value will receive no pheromone.

Example: Adding these arguments to the earlier ones:

(…full = 30, factors = c(‘Factor1’, ‘Factor2’), i.per.f = c(5,5), list.items = list(c(‘x1’, ‘x2’, ‘x3’, ‘x4’, ‘x5’, ‘x6’, ‘x7’, ‘x8’, ‘x9’, ‘x10’, ‘x11’, ‘x12’, ‘x13’, ‘x14’, ‘x15’), c(‘x16’, ‘x17’, ‘x18’, ‘x19’, ‘x20’, ‘x21’, ‘x22’, ‘x23’, ‘x24’, ‘x25’, ‘x26’, ‘x27’, ‘x28’, ‘x29’, ‘x30’)), ants = 20, evaporation = 90, steps = 100, max.run = 1000, min.CFI = 0.95, min.TLI = 0.95, max.RMSEA = 0.06)

***Interpreting the Results Output***

Both the antcolony.lavaan and antcolony.mplus functions create an antcolony object. If the antcolony object was saved as “short”, then entering short[[1]] will show the output for what the researcher is interested in: the fit statistics for the final short scale, its regression performance, and what items were selected to be included in it (coded in binary).

Simply entering short at the command line also gives this information, but with a lot of extra details about the algorithm’s performance that can be distracting. This information can be hard to read in the R console, and we recommend examining an output file that can be produced by the antcolony functions instead. (These functions can also produce a “summary file,” but this file is less helpful because it only provides information on the last ant in an iteration or when an ant fails to provide a valid solution; A. Raborn, personal communication, August 2, 2019).

The output file we are referring to is a feedback file that describes the performance of each ant (i.e., short form) at each iteration of the algorithm. To create this output file, one can add the following argument in either antcolony function: feedbackfile = “iteration.html”. Any file name can be used, but saving it as an html file is standard and makes the output easier to read than saving as a text file. The file is saved into the working directory and is organized in a format of blocks that looks like this:

run: 1 count: 1 ant: 1 step: 1
Fit Statistics: 0.994816223474683
GAMMA: 0.383807153702389 VAR.EXP: 0.149557437145322

run: 1 count: 1 ant: 1 step: 1
Fit Statistics: 0.994240248305203
GAMMA: 0.383807153702389 VAR.EXP: 0.149557437145322

run: 1 count: 1 ant: 1 step: 1
Fit Statistics: 0.00718761943100498
GAMMA: 0.383807153702389 VAR.EXP: 0.149557437145322

run: 1 count: 1 ant: 2 step: 1
Fit Statistics: 0.969414201341706
GAMMA: 0.377871237092234 VAR.EXP: 0.14238397689391

run: 1 count: 1 ant: 2 step: 1
Fit Statistics: 0.966015779268563
GAMMA: 0.377871237092234 VAR.EXP: 0.14238397689391

run: 1 count: 1 ant: 2 step: 1
Fit Statistics: 0.0169696447325381
GAMMA: 0.377871237092234 VAR.EXP: 0.14238397689391

Here, “run” is the current iteration of the algorithm, and “ant” is the number of the current short form being evaluated. Therefore, the output above contains information for the first two ants of the first iteration. For a given ant, the only thing that changes across its blocks are the fit statistics that are reported. The order the fit statistics are presented is determined by how they were specified in the antcolony function. Thus, for *k* fit statistics, there will be *k* blocks per ant. In the above output, three fit statistics were used, the CFI, TLI, and RMSEA. Each block also contains the (a) regression coefficient (“GAMMA”) and (b) the proportion of variance explained (“VAR.EXP”) for each short form. Finally, “count” and “step” are somewhat less important. “Count” indicates the number of iterations since the best short form was updated, and “step” refers to how many consecutive ants have selected the same short form.

**References**

Baker, F. (2001). *The basics of item response theory*. College Park, MD: ERIC Clearinghouse on Assessment and Evaluation.

Benjamin, R. G. (2012). Reconstructing readability: Recent developments and recommendations in the analysis of text difficulty. *Educational Psychology Review*, *24*(1), 63–88. https://doi.org/10.1007/s10648-011-9181-8

Brady, D. L., Brown, D. J., & Liang, L. H. (2017). Moving beyond assumptions of deviance: The reconceptualization and measurement of workplace gossip. *Journal of Applied Psychology*, *102*(1), 1–25. https://doi.org/10.1037/apl0000164

DeMars, C. (2010). *Item response theory*. Oxford: Oxford University Press.

Djurdjevic, E., Stoverink, A. C., Klotz, A. C., Koopman, J., da Motta Veiga, S. P., Yam, K. C., & Chiang, J. T. J. (2017). Workplace status: The development and validation of a scale. *Journal of Applied Psychology*, *102*(7), 1124–1147. https://doi.org/10.1037/apl0000202

Dorigo, M., & Stützle, T. (2004). *Ant Colony Optimization*. Cambridge, MA: MIT Press.

Edwards, M. C. (2009). An introduction to item response theory using the need for cognition scale. *Social and Personality Psychology Compass*, *3*(4), 507–529. https://doi.org/10.1111/j.1751-9004.2009.00194.x

Gigerenzer, G. (2004). Mindless statistics. *The Journal of Socio-Economics*, *33*(5), 587–606. https://doi.org/10.1016/j.socec.2004.09.033

Hinkin, T. R. (1998). A brief tutorial on the development of measures for use in survey questionnaires. *Organizational Research Methods*, *1*(1), 104–121. https://doi.org/10.1177/109442819800100106

Hu, L., & Bentler, P. M. (1999). Cutoff criteria for fit indexes in covariance structure analysis: Conventional criteria versus new alternatives. *Structural Equation Modeling, 6*, 1−55.

Janssen, A. B., Schultze, M., & Grotsch, A. (2015). Following the ants: Development of short scales for proactive personality and supervisor support by ant colony optimization. *European Journal of Psychological Assessment*.

Leite, W. L., Huang, I.-C., & Marcoulides, G. A. (2008). Item selection for the development of short forms of scales using an ant colony optimization algorithm. *Multivariate Behavioral Research*, *43*(3), 411–431. https://doi.org/10.1080/00273170802285743

Martinko, M. J., Mackey, J. D., Moss, S. E., Harvey, P., McAllister, C. P., & Brees, J. R. (2018). An exploration of the role of subordinate affect in leader evaluations. *Journal of Applied Psychology*, *103*(7), 738–752. https://doi.org/10.1037/apl0000302

Nunnally, J, C. (1978). *Psychometric theory*. New York: McGraw-Hill.

O’Connor, B. P. (2018). An illustration of the effects of fluctuations in test information on measurement error, the attenuation of effect sizes, and diagnostic reliability. *Psychological Assessment*, *30*(8), 991–1003. https://doi.org/10.1037/pas0000471

Orlitzky, M. (2011). How can significance tests be deinstitutionalized? *Organizational Research Methods*, *15*(2), 199–228. https://doi.org/10.1177/1094428111428356

Raborn, A., & Leite, W. (2018). *ShortForm: Automatic short form creation*. Retrieved from https://cran.r-project.org/package=ShortForm

Reise, S. P., Ainsworth, A. T., & Haviland, M. G. (2005). Item response theory: Fundamentals, applications, and promise in psychological research. *Current Directions in Psychological Science*, *14*(2), 95–101. https://doi.org/10.1016/B978-0-12-801504-9.00010-6

Schroeders, U., Wilhlem, O., & Olaru, G. (2016). Meta-heuristics in short scale construction: Ant colony optimization and genetic algorithm. *PLOS ONE*, *11*(11), 1–19. https://doi.org/10.5157/NEPS

Smith, G. T., Mccarthy, D. M., & Anderson, K. G. (2000). On the sins of short-form development. *Psycho-Oncology*, *12*(1), 102–111.
